# Correction: Stuckey et al. Why the Tsirelson Bound? Bub’s Question and Fuchs’ Desideratum. *Entropy* 2019, *21*, 692

**DOI:** 10.3390/e24050674

**Published:** 2022-05-11

**Authors:** William Stuckey, Michael Silberstein, Timothy McDevitt, Ian Kohler

**Affiliations:** 1 Department of Physics, Elizabethtown College, Elizabethtown, PA 17022, USA; kohleri@etown.edu; 2 Department of Philosophy, Elizabethtown College, Elizabethtown, PA 17022, USA; silbermd@etown.edu; 3 Department of Philosophy, University of Maryland, College Park, MD 20742, USA; 4 Department of Mathematical Sciences, Elizabethtown College, Elizabethtown, PA 17022, USA; mcdevittt@etown.edu

The authors wish to make the following correction to this paper [1]:

The four Bell basis states, Equation (30) on p. 13, represent one “unlike state” and three “like states”, not two “unlike states” and two “like states”. Thus, the analysis and Figure 8 on pages 13 and 14 for “unlike states” apply to only the first state in Equation (30), and the analysis and Figure 9 on pages 13 and 14 for “like states” apply to the other three states in Equation (30).

The conclusion is the same for “unlike states” and “like states”, so it is unaffected by this change in wording about which states in Equation (30) correspond to “unlike” and “like states” in the analysis. The original publication has been updated.
